# Hop flower extracts mitigate retinal ganglion cell degeneration in a glaucoma mouse model

**DOI:** 10.1038/s41598-020-78731-2

**Published:** 2020-12-10

**Authors:** Tomoko Hasegawa, Hanako O. Ikeda, Sachiko Iwai, Norio Sasaoka, Akira Kakizuka, Akitaka Tsujikawa

**Affiliations:** 1 Department of Ophthalmology and Visual Sciences, Kyoto University Graduate School of Medicine, 54 Kawahara-cho, Shogoin, Sakyo-ku, Kyoto, Kyoto 606-8507 Japan; 2 Research Fellow of Japan Society for the Promotion of Science, Tokyo, Japan; 3 Laboratory of Functional Biology, Kyoto University Graduate School of Biostudies, Konoe-cho, Yoshida, Sakyo-ku, Kyoto, Kyoto 606-8501 Japan

**Keywords:** Optic nerve diseases, Eye diseases

## Abstract

In glaucoma, retinal ganglion cells degenerate progressively, leading to visual field loss and blindness. Presently, the only treatment strategy for glaucoma is lowering the intraocular pressure. However, there are cases in which patients develop progressive visual field loss even though their intraocular pressures are within normal ranges. Therefore, the development of novel therapeutic strategies is an urgent endeavor. Besides high intraocular pressure, several other factors have been proposed to be associated with glaucoma progression, e.g., myopia, blood flow impairment, and amyloid β accumulation. We have previously reported that hop flower extracts possess γ-secretase inhibitory activities and reduce amyloid β deposition in the brains of Alzheimer’s disease model mice. In the current study, we showed that administration of hop flower extracts to glutamate-aspartate transporter (GLAST) knockout mice, the glaucoma model mice, attenuated glaucomatous retinal ganglion cell degeneration. Preservation of retinal ganglion cells in hop flower extract-administered mice was confirmed using optical coherence tomography, confocal scanning laser ophthalmoscopy, and retinal flatmount and histological evaluations. Hop flower extracts are, therefore, deemed a possible candidate as a novel therapeutic agent to treat glaucoma.

## Introduction

In glaucoma, retinal ganglion cells degenerate gradually, leading to progressive visual field loss and ultimately blindness. Presently, reduction of intraocular pressure is the only treatment strategy for glaucoma; this process can slow the progression of disease in approximately 80% of patients with glaucoma^1,2^. However, there have been cases of patients who develop progressive visual field loss, despite sufficiently lowered intraocular pressures^1,3^. While age and intraocular pressure are the known risk factors for glaucoma progression^4^, other possible risk factors such as myopia and blood flow impairment have also been reported^4–8^. In addition, a possible relationship between amyloid β (Aβ) deposition and glaucoma has drawn increasing attention^9–11^. Aβ accumulation, which causes Alzheimer’s disease, has been reported to cause apoptotic cell death of retinal ganglion cells in glaucoma^9,11^. Furthermore, it has been reported that elevated intraocular pressure increases Aβ accumulation in the retina of glaucoma animal models^9–11^.

We have previously shown that hop flower extracts exhibit γ-secretase inhibitory activities and inhibit Aβ production in cultured cells and in Alzheimer’s disease model mice with mutated amyloid precursor protein (APP), in which the C-terminal portion of human APP with the Indiana mutation (V717F) was expressed in neuronal cells driven by the neuron-specific enolase (NSE) promoter^12^. Specifically, oral administration of hop flower extracts to the Alzheimer’s disease model mice decreased Aβ deposition in their brains and mitigated their memory impairment^12^.

Glutamate-aspartate transporter (GLAST) knockout mice have been widely used as a model for normal tension glaucoma, which is characterized by chronic retinal ganglion cell death and optic nerve degeneration without elevated intraocular pressure^13^. GLAST is a major glutamate transporter in the retina. In GLAST knockout mice, retinal ganglion cell damage is induced by chronic excitotoxicity due to excessive glutamate. Compared to wild-type mice, GLAST knockout mice show fewer retinal ganglion cells, thinner optic nerves, lesser ganglion cell complex thickness as measured using OCT, and deteriorating retinal function as measured using electroretinography^13,14^. Retinal ganglion cell degeneration in GLAST (−/−) mice is more severe than that in GLAST (+/−) mice.

In the current study, we examined if administration of hop flower extracts attenuates retinal ganglion cell degeneration in GLAST knockout mice.

## Results

### Ganglion cell complex thickness of treated and non-treated mice

**Figure 1:**
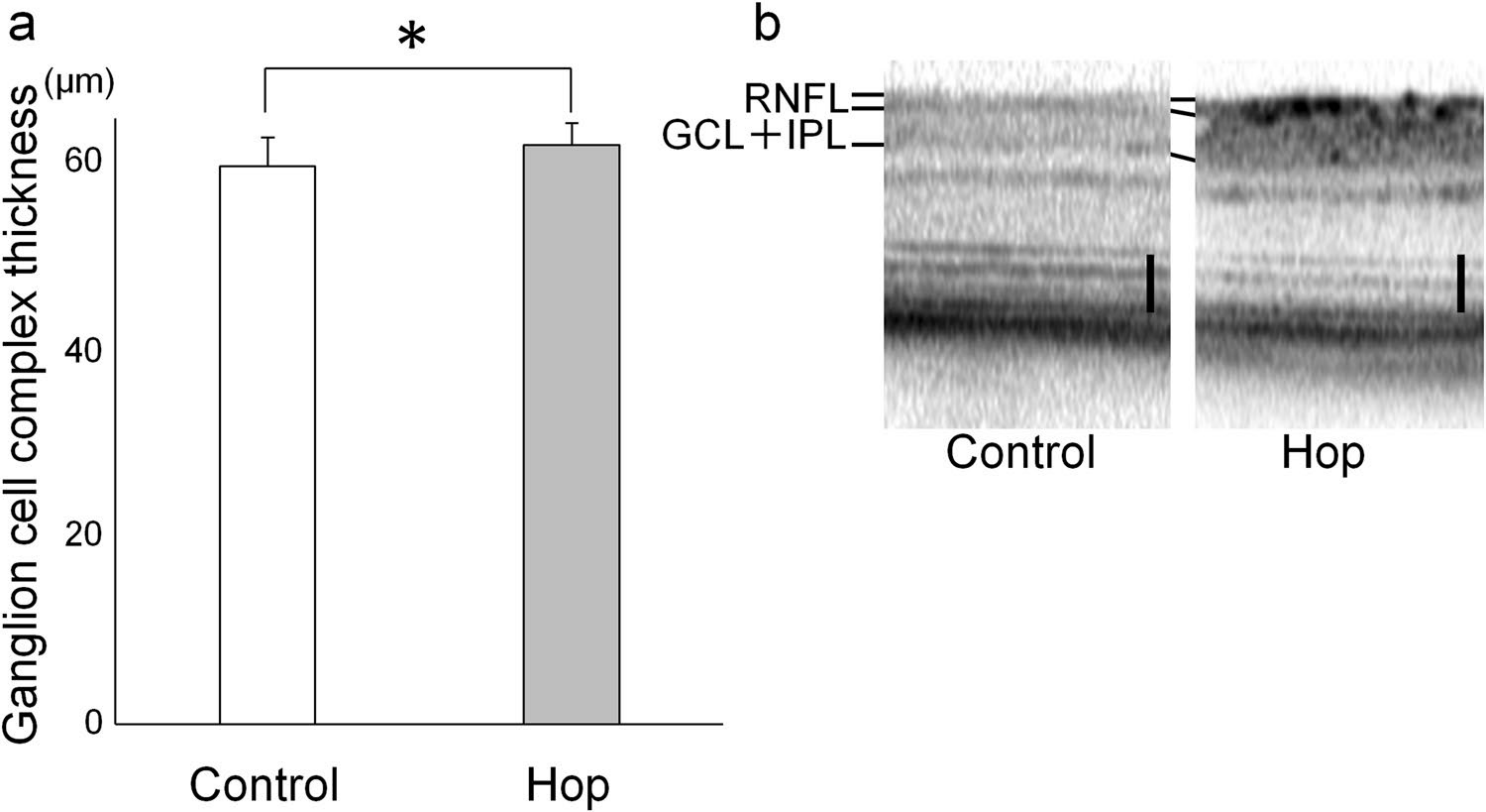
Prevention of ganglion cell complex thinning in GLAST (−/−) mice. (**a**) Ganglion cell complex thickness in 12-week-old GLAST (−/−) mice administered with hop extracts or water as a control. Ganglion complex thickness was measured as the sum of the retinal nerve fiber layer (RNFL), the ganglion cell layer (GCL), and the inner plexiform layer (IPL). *p ≤ 0.05, unpaired *t*-test, n = 16 and 10 eyes in the hop-treated and control groups, respectively. (**b**) A spectral domain optical coherence tomography (SD-OCT) image of 12-week-old GLAST (−/−) mice administered with hop extracts or water as a control. The black bars represent 50 μm.

The effect of hop flower extracts was first examined in GLAST (−/−) mice^13^. Ganglion cell complex thickness, which becomes thin in glaucoma, was evaluated as the sum of the thickness of the retinal nerve fiber layer, the retinal ganglion cell layer, and the inner plexiform layer. Although ganglion cell complex thickness did not show any significant differences between groups at 4 and 5 weeks of age (70.6 ± 5.0 and 68.8 ± 5.3 μm in 4-week-old GLAST (−/−) mice with and without hop extracts administration, and 64.3 ± 3.9 and 63.3 ± 4.2 μm in 5-week-old GLAST (−/−) mice with and without hop extracts administration; n = 16 and 10 eyes, respectively; p = 0.20 and 0.27, respectively; unpaired *t*-test), ganglion cell complex was thicker in 8- and 12-week-old GLAST (−/−) mice administered with hop extracts (hop-treated group) than in non-treated mice (control group) (65.0 ± 3.2 and 62.4 ± 3.1 μm in 8-week-old GLAST (−/−) mice, and 62.1 ± 2.4 and 59.9 ± 3.1 μm in 12-week-old GLAST (−/−) mice, n = 16 and 10 eyes, respectively; p = 0.026 and 0.025 respectively, unpaired *t*-test) (Fig. 1). There was no significant difference in the thickness of outer retinal layers between the groups (photoreceptor thickness—measured as the sum of the outer nuclear layer, photoreceptor myoid zone, photoreceptor ellipsoid zone, and outer segment layer thickness—was 90.2 ± 4.9 (hop-treated group, n = 16 eyes) and 89.1 ± 1.9 μm (control group, n = 10 eyes) in 12-week-old GLAST (-/-) mice; p = 0.51; unpaired *t*-test).

### Retinal ganglion cell numbers of treated and non-treated mice

The effect of hop flower extract treatment in GLAST (+/−) mice over a longer time period was evaluated. GLAST knockout mice were crossed with Thy1-CFP mice to produce GLAST (+/−):Thy1-CFP mice^14^, in which retinal ganglion cells express cyan fluorescent protein (CFP)^15,16^. The number of retinal ganglion cells was lower in 12- and 18-month-old GLAST (+/−):Thy1-CFP mice than in GLAST-intact [WT; GLAST (+/+):Thy1-CFP] mice of the same age (Suppl Fig. S1).

**Figure 2:**
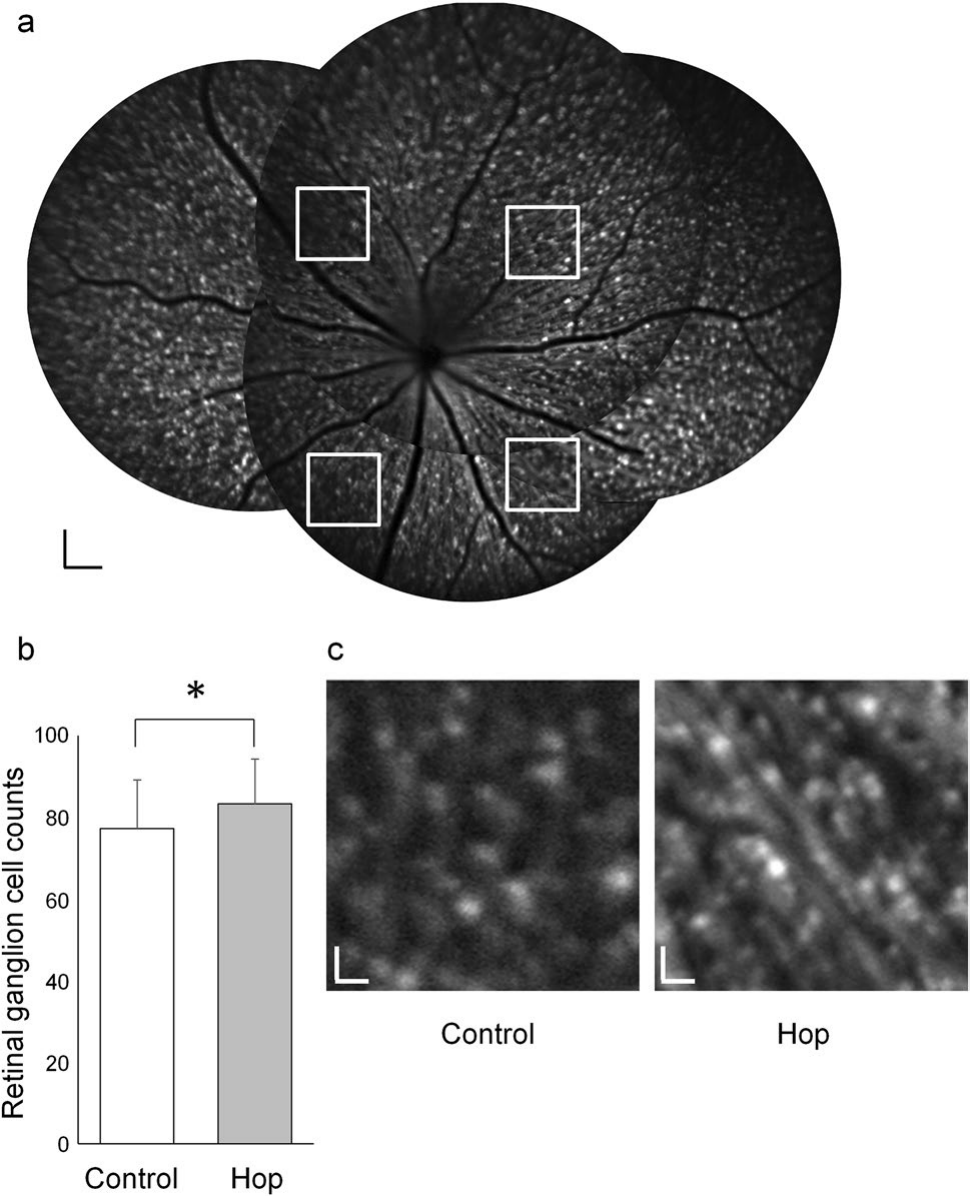
Prevention of retinal ganglion cell decrease in GLAST (+/−) mice administered with hop extracts, shown on SLO images. (**a**) Montage scanning laser ophthalmoscope (SLO) image of 12-month-old GLAST (+/−):Thy1-CFP mice. CFP positive retinal ganglion cells were manually counted in a masked fashion within 186 μm squares (white squares), at a distance of 456 μm from the disc center, in four directions. (**b**) Ganglion cell counts of 12-month-old GLAST (+/−):Thy1-CFP mice administered with hop extracts or water as a control. *p ≤ 0.05, unpaired *t*-test, n = 26 and 24 eyes in the hop-treated and control groups, respectively. (**c**) Representative images of counted squares of 12-month-old GLAST (+/−):Thy1-CFP mice administered with hop extracts or water as a control. Bars represent 100 µm in (**a**) and 20 µm in (**c**).

**Figure 3:**
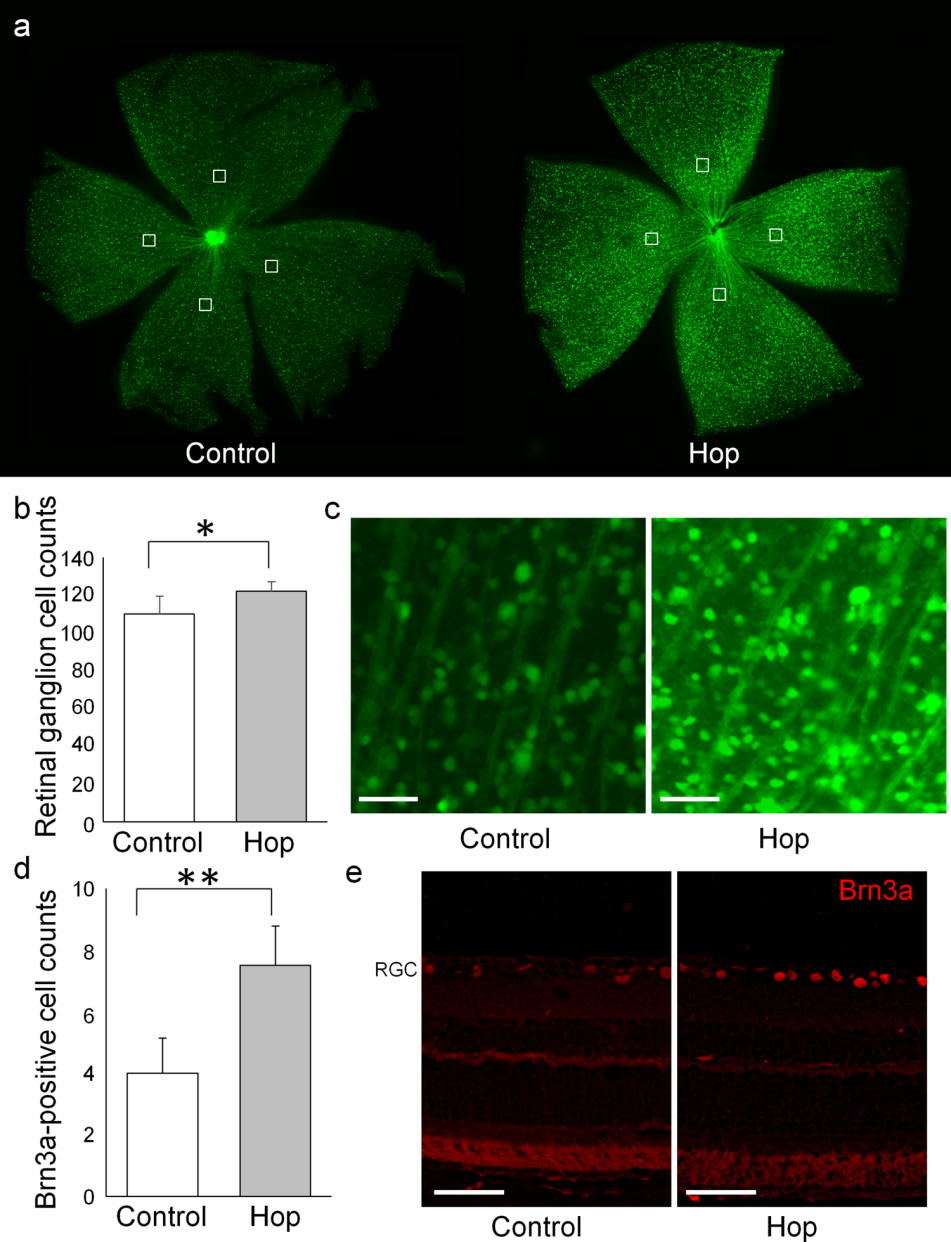
Prevention of retinal ganglion cell decrease in GLAST (+/−) mice administered with hop extracts on retinal flatmounts and immunohistochemical evaluation. (**a**–**c**) Retinal flatmounts of 12-month-old GLAST (+/−):Thy1-CFP mice administered hop extracts or water as a control. CFP positive retinal ganglion cells were manually counted in a masked fashion within 250 μm squares (white squares in **a**), at a distance of 1200 μm from the disc center, in four directions. (**b**) Ganglion cell counts of 12-month-old GLAST (+/−):Thy1-CFP mice administered with hop extracts or water as a control. *p ≤ 0.05, unpaired *t*-test, n = 6 eyes, respectively. (**c**) Representative images of counted squares of hop-treated or control 12-month-old GLAST (+/−):Thy1-CFP mice. (**d**, **e**) Immunohistochemical analysis using anti-brain-specific homeobox/POU domain protein 3A (Brn3a) antibody in hop-treated or control 12-month-old GLAST (+/−) mice retinas. (**d**) Brn3a-positive retinal ganglion cells in the retinal ganglion cell layer (RGC) were counted in a masked fashion within a distance between 1000 and 1100 μm from the disc center. **p ≤ 0.01, unpaired *t*-test, n = 4 eyes, respectively. (**e**) Representative images of sections immunostained with anti-Brn3a antibody. Bars represent 50 µm in (**c**) and (**e**).

Short-wavelength scanning laser ophthalmoscopy (scSLO) images revealed that the retinal ganglion cell numbers of 12-month-old GLAST (+/−):Thy1-CFP mice in the hop-treated group were greater than those in the control group (83.3 ± 11.1 and 77.2 ± 12.0, n = 26 and 24 eyes, respectively; p = 0.035, unpaired *t*-test) (Fig. 2). The retinal ganglion cell numbers of 12-month-old GLAST (+/−):Thy1-CFP mice on retinal flatmounts were also greater in the hop-treated group than in the control group (122.0 ± 5.3 and 110.0 ± 9.6, respectively; n = 6 eyes each; p = 0.011, unpaired *t*-test) (Fig. 3a–c). Immunohistochemical evaluation of retinal samples from 12-month-old GLAST (+/−) mice using anti-brain-specific homeobox/POU domain protein 3A (Brn3a) antibody revealed a greater number of retinal ganglion cells in the hop-treated group than that in the control group (7.5 ± 1.3 and 4.0 ± 1.2 cells, respectively; n = 4 eyes each; p = 0.007; unpaired *t*-test) (Fig. 3d,e).

### Optic nerve cross sections of treated and non-treated mice

**Figure 4:**
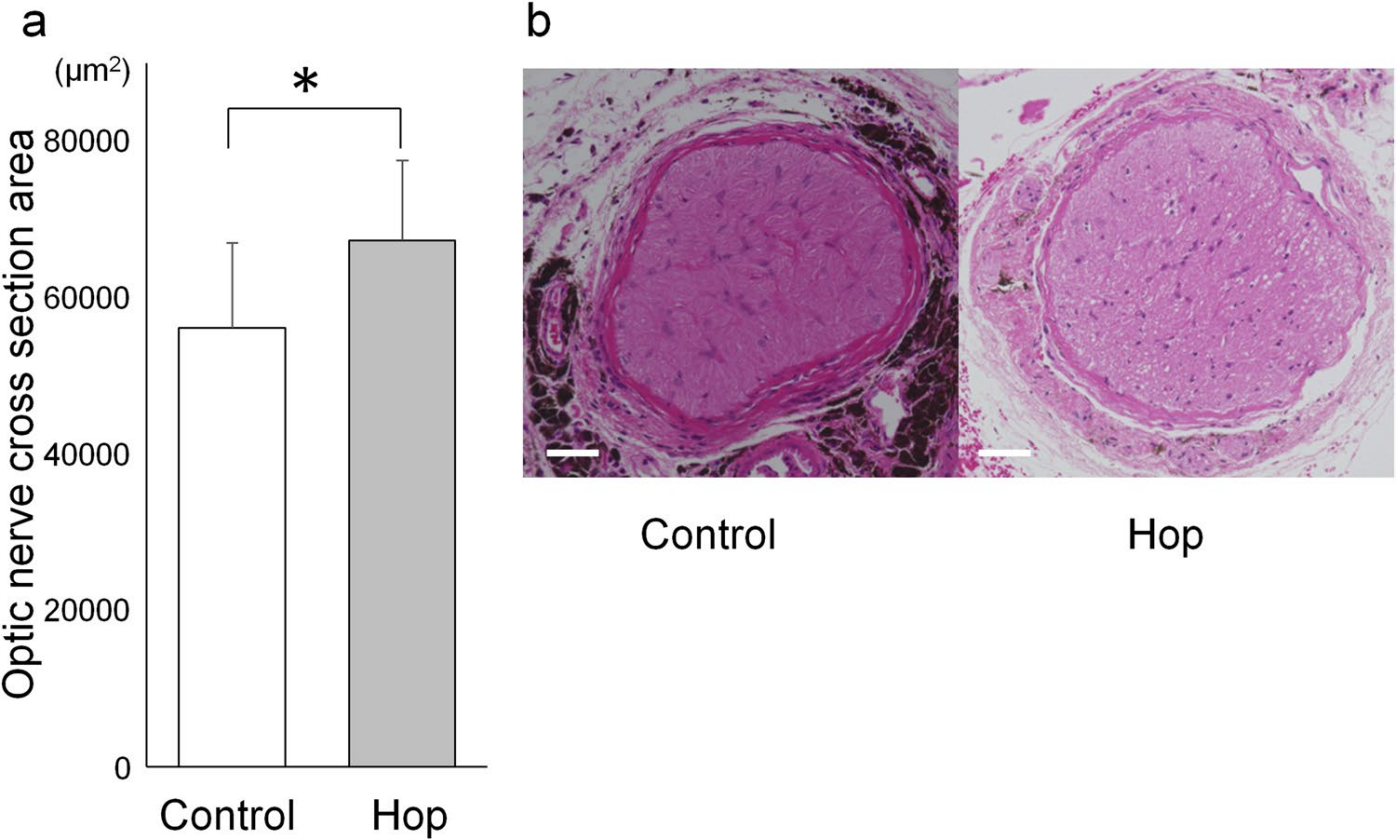
Prevention of optic nerve thinning in GLAST (+/−) mice administered with hop extracts. (**a**) Optic nerve cross section area of 18-month-old GLAST (+/−):Thy1-CFP mice administered with hop extracts or water as a control. *p ≤ 0.05, unpaired *t*-test, n = 6 and 8 eyes in the hop-treated and control groups, respectively. (**b**) Images of the optic nerve cross sections of 18-month-old GLAST (+/−):Thy1-CFP mice administered with hop extracts or water as a control, indicating the median areas. Bars represent 50 µm.

The area of optic nerve cross sections of 18-month-old GLAST (+/−):Thy1-CFP mice was larger in the hop-treated group than in the control group (67,320 ± 10,221 and 56,140 ± 10,887 µm^2^, n = 6 and 8 eyes, respectively; p = 0.037, unpaired *t*-test) (Fig. 4).

### Amyloid β immunostaining of treated and non-treated mice

**Figure 5:**
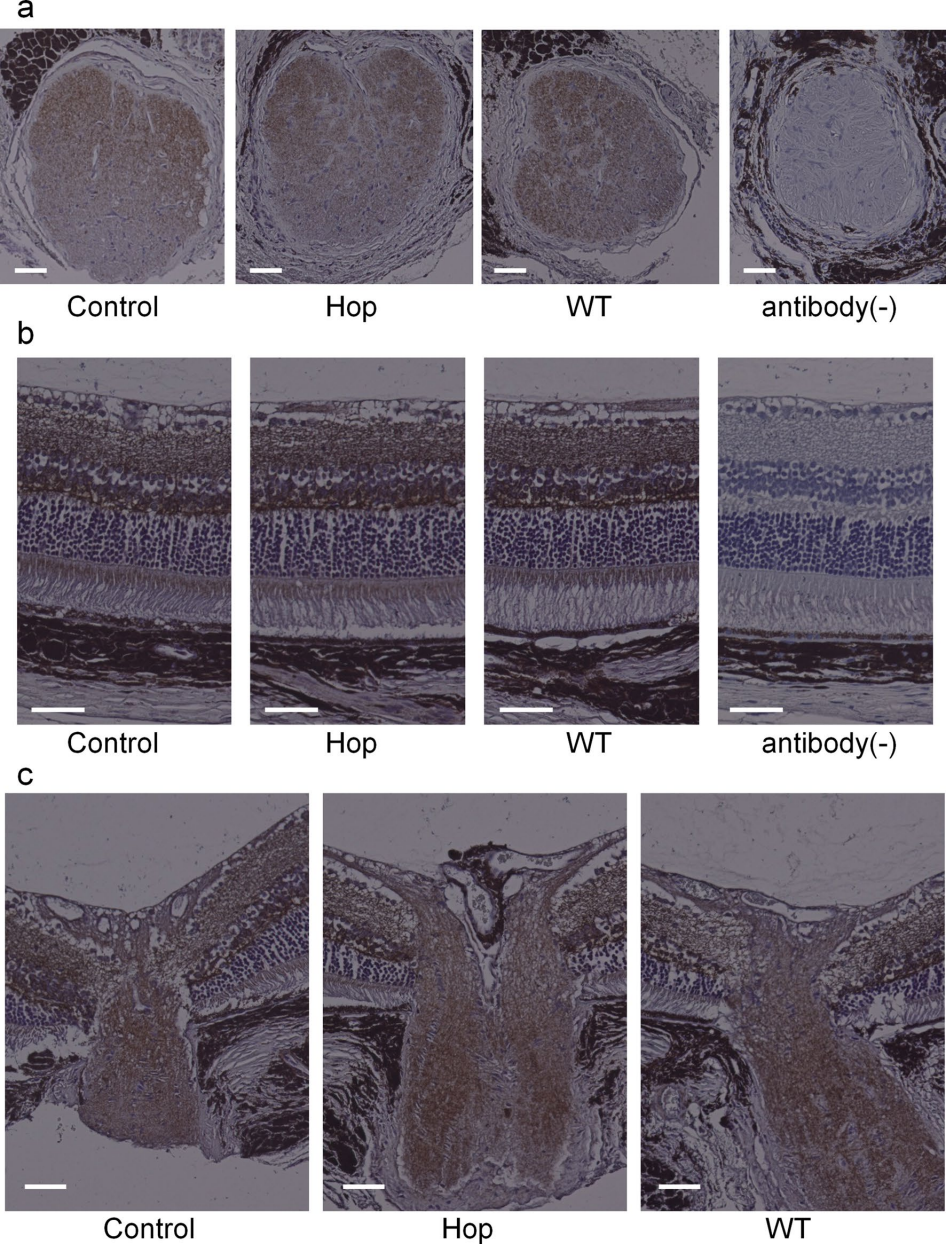
Amyloid β immunostaining of optic nerve cross sections and retinal sections in GLAST (+/−) mice treated with or without hop extracts and GLAST (+/+, wild type) mice. Optic nerve cross sections and retinal sections of 18-month-old GLAST (+/−) mice administered with hop extracts or water as a control and GLAST wild type (WT) mice were stained with or without the presence of anti-amyloid β antibody. (**a**) Optic nerve cross sections. (**b**) Retinal sections. (**c**) Optic nerve vertical sections. Bars represent 50 µm in (**a**–**c**).

The immunostaining intensity of Aβ in optic nerve cross sections of 18-month-old GLAST (+/−):Thy1-CFP mice did not differ between the hop-treated and the control groups (staining intensity of the cross sections were 0.65 ± 0.05 and 0.65 ± 0.03, respectively; n = 5 eyes each; p = 0.93, unpaired *t*-test) (Fig. 5).

## Discussion

In this study, we showed that administration of hop flower extracts attenuates retinal ganglion cell degeneration in GLAST knockout mice, a glaucoma mouse model.

Increased occurrence of glaucoma and glaucomatous retinal changes in patients with Alzheimer’s disease have been widely reported^17–20^. Therefore, Alzheimer’s disease, caused by accumulation of Aβ, is considered to be associated with glaucoma. Besides the evidence of Aβ accumulation in experimental animal models of glaucoma with ocular hypertension^9–11^, it has also been reported that Aβ induces apoptotic retinal ganglion cell death^9^. However, studies have also found glutamate-induced alteration in the amyloid precursor protein processing and Aβ production^21,22^. Moreover, it was reported that GLAST protein expression is reduced in the brains of patients with Alzheimer’s disease in the early clinical stages^22^. In the current study, we administered GLAST knockout glaucoma model mice with hop flower extracts, which have been shown to possess γ-secretase inhibitory activities. The administration of hop flower extracts attenuated retinal ganglion cell degeneration, albeit the differences were not big (Figs. 1–4).

The current study has several limitations. First, the number of animals available was limited because of the difficulty in breeding GLAST knockout mice, particularly, GLAST (−/−) mice. The powers of the experiments were determined using the G* power 3.1.9.2 tool^23^ and was calculated to be 0.62 for ganglion cell complex thickness of GLAST (−/−) mice (Fig. 1a), 0.57 for ganglion cell measurement on SLO of GLAST (+/−) mice (Fig. 2b), 0.80 for ganglion cell measurement on flatmounts of GLAST (+/−) mice (Fig. 3b), and 0.58 for optic nerve cross sections measurement of GLAST (+/−) mice (Fig. 4a). Based on the calculated G power values, the sample size was deemed to be appropriate for the flatmounts; however, in future studies, it would be better to conduct these experiments with a larger number of animals. If more animals could be examined, the differences would be clearer. Second, the assessment of the thickness of ganglion cell complex on OCT images (Fig. 1) can be affected not only by retinal ganglion cells but also by other cells, such as bipolar, amacrine, and horizontal cells. Alternatively, we considered CFP-positive cells of Thy1-CFP mice as retinal ganglion cells (Figs. 2 and 3). It has been reported that 97% of the CFP-positive cells are retinal ganglion cells, while 73% of the retinal ganglion cells express CFP in Thy1-CFP mice^16^. This observation implies that on the assessment of CFP-positive cells, some of the retinal ganglion cells, those without CFP fluorescence, might be missed. We also confirmed the maintenance of retinal ganglion cells in the hop-treated group using immunohistochemical analysis with an anti-Brn3a antibody. Lastly, the mechanisms by which hop flower extract attenuated retinal ganglion cell degeneration remains unclear. Regarding Aβ deposition, we could not detect extract-related reduction of Aβ in the retina and optic nerves on immunohistochemical analysis when the hop-treated mice were compared with the control mice (Fig. 5). However, possible changes in Aβ levels, too small to be detected, may be present. Since glutamate levels at the synapse and Aβ levels are altered synergistically, the possibility that small concentration changes could influence Alzheimer’s disease state has been discussed^22^. In addition, there is a possibility that hop flower extracts attenuate retinal ganglion cell degeneration through mechanisms other than Aβ reduction, which we could not identify in this study. In fact, the mechanisms underlying glaucoma progression are still not fully understood and a considerable number of genes have been implicated in the pathogenesis of glaucoma^24^.

In the current study, functional evaluation of hop flower extracts was performed on 4-month-old GLAST (−/−) and 12-month-old GLAST (+/−) mice using electroretinography; the amplitudes of positive scotopic threshold response reflecting the inner retinal function^25^ were measured. The amplitudes were higher in hop-treated mice than in control mice, although the differences did not reach a significance level due to the large differences between individual responses and the relatively small number of animals in the study (p = 0.17; 10.6 ± 9.0 and 6.9 ± 3.1 μV, n = 16 and 12 eyes, respectively, with a stimulus intensity of -4.57 log cds/m^2^, for 4-month-old GLAST (−/−) mice, and p = 0.22; 46.2 ± 27.3, and 40.0 ± 23.7 μV, n = 21 eyes each, with a stimulus intensity of − 4.12 log cds/m^2^ for 12-month-old GLAST (+/−) mice.

In conclusion, hop flower extracts attenuated retinal ganglion cell degeneration in a glaucoma mouse model and therefore should be considered in the development of novel therapeutic agents for treating glaucoma.

## Methods

### Experimental animals

This study was conducted in accordance with the Association for Research in Vision and Ophthalmology (ARVO) Statement for the Use of Animals in Ophthalmic and Vision Research. All protocols were approved by the Institutional Review Board of Kyoto University Graduate School of Medicine (MedKyo 14,213, 15,531, 16,501, 17,272, 19,298, 180,282, and 170,457). B6.Cg-Tg(Thy1-CFP)23Jrs/J mice, in which CFP is expressed in retinal ganglion cells under the control of the Thy1 promoter^15,16^, were obtained from the Jackson Laboratory (Bar Harbor, ME, USA). GLAST knockout mice^13^ were provided as a gift from Dr. Koichi Tanaka (Tokyo Medical and Dental University). GLAST knockout mice and Thy1-CFP mice were crossed to produce GLAST (+/−):Thy1-CFP mice^14^. GLAST (+/+):Thy1-CFP mice were compared to GLAST (+/−):Thy1-CFP mice at 12 and 18 months of age. GLAST (+/−):Thy1-CFP mice and GLAST (−/−) mice were assigned to either the hop-treated group or the control group. All mice were maintained in a 14-h light/10-h dark cycle and were fed ad libitum. Before imaging, mice were anesthetized by an intramuscular injection of a ketamine (70 mg/kg)/xylazine (14 mg/kg) mixture, and their pupils were dilated using tropicamide and phenylephrine eye drops (0.5% each).

### Administration of hop flower extracts

Hop flower extracts were prepared as described previously^12^. The Bligh-Dyer method was performed, and the collected lipid-soluble fraction, which has γ-secretase inhibitory activities, was used in the experiment (referred to as hop extracts). The hop extracts were suspended in water using an ultrasonic dissolver (1 g/L for oral administration and 2 g/L for peritoneal administration).

Seven-day-old GLAST (−/−) mice were assigned to either the hop-treated group or control group. Hop-treated group mice were administered hop extracts (0.02 g/kg/day) intraperitoneally from 7 days to 2 months of age, after which they had ad libitum access to oral medication in the form of water containing 1 g/L of hop extracts. Control group mice were subjected to intraperitoneal administration of saline from 7 days to 2 months of age and had ad libitum access to water after reaching 2 months of age. For hetero GLAST-deficient mice experiments, 1-month-old GLAST (+/−):Thy1-CFP mice were assigned to either the hop-treated or the control groups. The hop-treated mice had ad libitum access to water containing 1 g/L of hop extracts. For oral ad libitum administration of hop extracts, water decrement in the water bottles was measured; the administration dosage was about 0.2 g/kg/day, with an assumption that the mice drank all of the decremented amount.

### OCT and SLO image acquisition

For imaging, a custom made short-wavelength confocal scanning laser ophthalmoscope (scSLO) that was optimized for CFP imaging was used. As previously described, this combined system included a laser with a short wavelength (445 nm) and the speckle noise-reduced spectral domain optical coherence tomography (SD-OCT) with the eye-tracking function based on SPECTRALIS HRA + OCT (*Multiline OCT*; Heidelberg Engineering, Heidelberg, Germany)^26^. A 25-diopter adaptor lens was placed on the objective lens of the *Multiline OCT* to focus on the mouse retina; a poly methyl methacrylate (PMMA) contact lens (Nichicon, Osaka, Japan), which is optimal for mice, was placed on the corneas, as previously described^26^, to prevent anesthesia-induced cataract progression. All lateral dimensions shown using the system software (originally scaled for human eyes) were converted to those of mouse eyes by multiplying the values by 0.122^26^. For SD-OCT imaging, a circular scan around the optic disc (with a diameter of 0.42 mm) was performed, and 16 images were used for averaging. For scSLO imaging, areas around the optic disc were scanned, and 30 images per scan area were averaged. GLAST (−/−):Thy1-CFP mice were imaged using SD-OCT at 4, 5, 8, and 12 weeks of age. GLAST (+/−):Thy1-CFP mice were imaged using scSLO at 12 months of age.

### OCT and SLO image analyses

The thickness of the ganglion cell complex, which was evaluated as the sum of the retinal nerve fiber layer, the retinal ganglion cell layer, and the inner plexiform layer, was measured on the circular OCT images using the built-in software of the SPECTRALIS HRA-OCT system.

The SLO images were merged by matching the vessels and the CFP positive retinal ganglion cell patterns using Adobe Photoshop (ver. 12.1) to produce a montage of SLO images. CFP-positive retinal ganglion cells were manually counted in a masked fashion within areas of 186 μm squares, at a distance of 456 μm from the disc center, in four directions (Fig. 2a). The counted numbers in the four directions were then averaged.

### Retinal flatmount

Eyeballs of 12-month-old GLAST (+/−):Thy1-CFP mice were enucleated after pentobarbital overdose and fixed in 4% paraformaldehyde for 1 h. The cornea, lens, uvea, and sclera were removed and retinal flatmounts were made^14,27^. The retinal flatmounts were observed and photographed under an optical microscope (BZ-9000; Keyence, Osaka, Japan). CFP-positive retinal ganglion cells were counted manually in a masked fashion within areas of 250 μm squares, at a distance of 1200 μm from the disc center, in four directions^27^ (Fig. 3a). The counted numbers in the four directions were then averaged.

### Histologic and immunostaining optic nerve evaluations

The eyeballs and linked optic nerves of 18-month-old GLAST (+/−):Thy1-CFP mice were extracted after pentobarbital overdose, fixed in 4% paraformaldehyde at 4 °C overnight, and then embedded in paraffin. Optic nerve cross sections were obtained by cutting 6-μm serial sections^14,27^, and these sections were stained with hematoxylin and eosin (HE). For immunostaining with an anti-amyloid β antibody, optic nerve cross sections and retinal sections were treated with 0.3% hydrogen peroxide to block endogenous peroxidase activities and washed with TBST (Tris-buffered saline with 0.3% Triton X). After incubating in TBST for 30 min with 10% normal goat serum and 1% bovine serum albumin, sections were incubated overnight with anti-amyloid β antibody (2.5 μg/mL, 18584, Immuno-Biological Laboratories, Fujioka, Gunma, Japan). The sections were then washed with TBST and incubated for 60 min with biotinylated goat anti-rabbit antibody (1:250, BA-1000, Vector Laboratories, Burlingame, CA, USA). After washing with TBST, the sections were incubated in ABC solution (Vectastain PK-4000, Vector Laboratories, Burlingame, CA, USA), re-washed in TBST, stained with DAB solution (D006, DOJINDO Laboratories, Kamimashiki, Kumamoto, Japan), washed in water, and then stained with hematoxylin. For immunostaining with the anti-Brn3a antibody, the sections were incubated overnight with the anti-Brn3a antibody (1:100, MAB1585, Chemicon, Temecula, CA, USA). After washing, the sections were incubated for 60 min with an Alexa Fluor 594-conjugated anti-mouse antibody (1:1000, A21203, Thermo Fisher Scientific, Waltham, MA, USA). The stained sections were observed and photographed under an optical microscope (BZ-9000; Keyence, Osaka, Japan). The area of the optic nerve cross sections and the staining intensity of the immuno-stained sections were measured using BZ-II Analyzer software (Keyence, Osaka, Japan). The Brn-3a positive cells were manually counted in a masked fashion within a distance of 1000–1100 μm from the optic disc center using BZ-II Analyzer software (Keyence, Osaka, Japan).

### Statistical analyses

Data are presented as mean ± standard deviation. An unpaired *t*-test was used to compare parameters between treated and untreated mice. Statistical significance was defined as p < 0.05.

## Supplementary Information

Supplementary Figure S1.

## Data Availability

All data generated or analyzed during this study are included in this published article and in the supplementary information.
