# Supplementary material for: Hop flower extracts mitigate retinal ganglion cell degeneration in a glaucoma mouse model
Source: Sci Rep. 2020 Dec 10;10:21653. doi: 10.1038/s41598-020-78731-2 (PMC7730440; doi:10.1038/s41598-020-78731-2)
Supplement: Supplementary file 1 — Supplementary Figure S1. [file 41598_2020_78731_MOESM1_ESM.pdf]

Supplementary information

Hop flower extracts mitigate retinal ganglion cell degeneration in a glaucoma mouse model

Tomoko Hasegawa, Hanako Ohashi Ikeda, Sachiko Iwai, Norio Sasaoka, Akira Kakizuka, Akitaka Tsujikawa

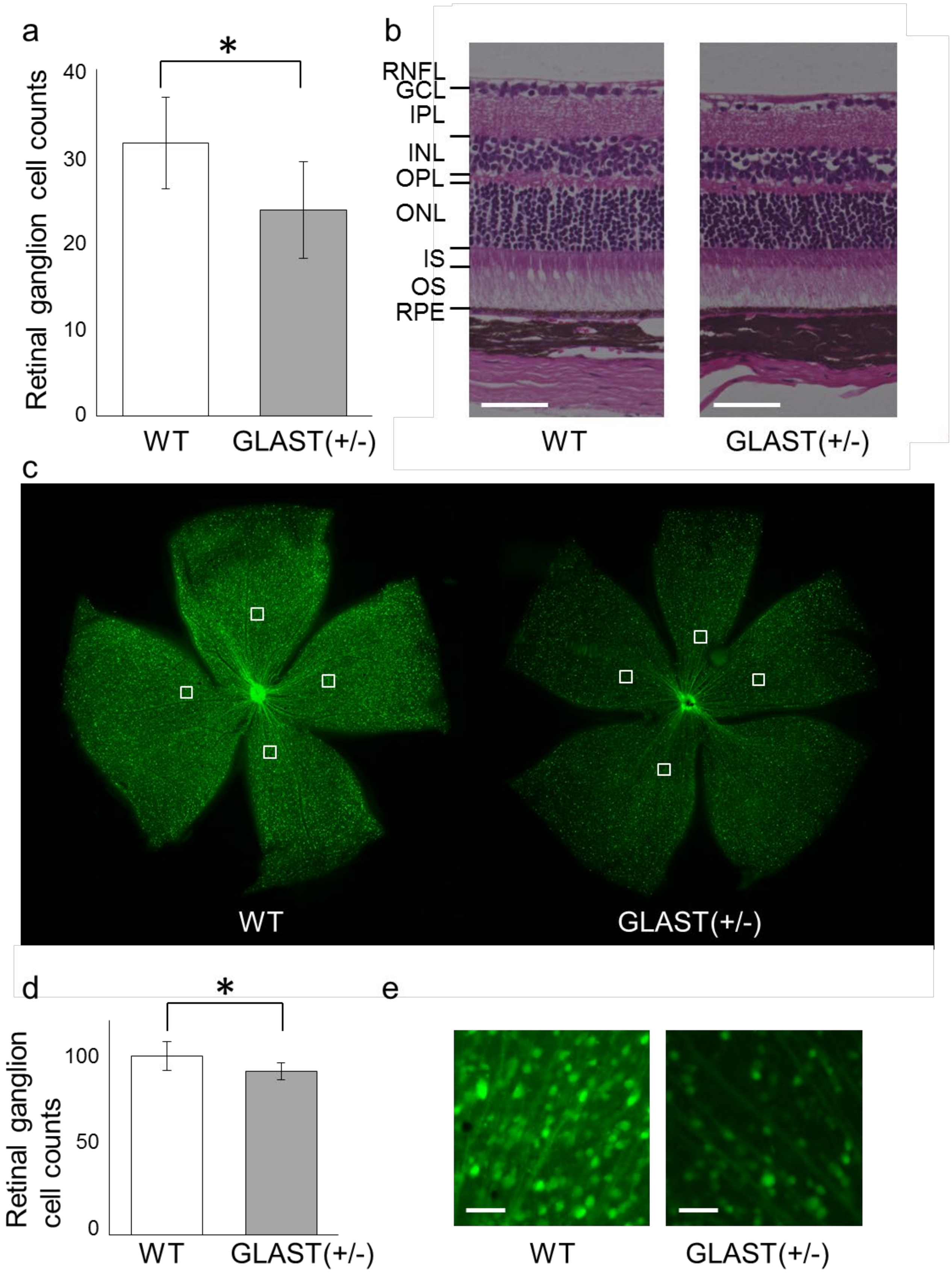
a
*
Retinal ganglion cell counts
40
30
20
10
0
WT
GLAST(+/-)
b
RNFL
GCL
IPL
INL
OPL
ONL
IS
OS
RPE
WT
GLAST(+/-)
c
WT
GLAST(+/-)
d
*
Retinal ganglion cell counts
100
50
0
WT
GLAST(+/-)
e
WT
GLAST(+/-)

Figure S1. Retinal ganglion cell counts in GLAST (+/-) mice and wild type mice. (**a**) Retinal ganglion cell counts of 12-month-old GLAST (+/-):Thy1-CFP mice and GLAST wild type GLAST (+/+):Thy1-CFP mice. Before enucleation, the superior portion of the eyeballs were marked with a suture placed on the edge of the superior conjunctiva. The eyeballs were extracted after pentobarbital overdose, fixed in 4% paraformaldehyde at 4 °C overnight, and then embedded in paraffine. Serial 6-μm paraffin-embedded sections were cut through the suture and at the point of insertion of the optic nerve. Sections including the center of the optic nerve head were stained with hematoxylin and eosin, observed and photographed under an optical microscope (BZ-9000; Keyence, Osaka, Japan). The retinal ganglion cell numbers (represented by the nucleus numbers at the retinal ganglion cell layer) were counted at a distance between 500 μm and 800 μm away from the edge of the optic nerve. *p = < 0.05, unpaired *t*-test, n = 4 eyes each. (**b**) Images of the vertical retinal sections of 12-month-old GLAST (+/-):Thy1-CFP mice and GLAST wild type GLAST (+/+):Thy1-CFP mice. Bars represent 50 μm. Abbreviations: RNFL, retinal nerve fiber layer; GCL, ganglion cell layer; IPL, Inner plexiform layer; INL, Inner nuclear layer; OPL, Outer plexiform layer; ONL, outer nuclear layer; IS, inner segment of the photoreceptor cell; OS, Outer segment of the photoreceptor cell; and RPE, Retinal pigment epithelium. (**c**) Retinal flatmounts of 18-month-old GLAST (+/-):Thy1-CFP mice and GLAST wild type GLAST (+/+):Thy1-CFP mice. CFP positive retinal ganglion cells were manually counted in a masked fashion within 250 μm squares (white squares), at a distance of 1,200 μm from the disc center, in four directions. (**d**) Retinal ganglion cell counts of 18-month-old GLAST (+/-):Thy1-CFP mice and GLAST wild type GLAST (+/+):Thy1-CFP mice. *p = < 0.05, unpaired *t*-test, n = 5 eyes each. (**e**) Representative images of counted squares of 18-month-old GLAST (+/-):Thy1-CFP and GLAST wild type GLAST (+/+):Thy1-CFP mice. Bars represent 50 μm in (**b**) and (**c**).
